# Effective real-time self-rehabilitation exercise monitoring and correctness system for low back pain management

**DOI:** 10.1038/s41598-026-51088-8

**Published:** 2026-05-07

**Authors:** Dilliraj Ekambaram, Vijayakumar Ponnusamy, C. S. Asha, K Suganthi

**Affiliations:** 1 Department of Electronics and Communication Engineering, SRM Institute of Science and Technology, Kattankulathur, Chengalpattu, 603203 Tamil Nadu India; 2 Manipal Institute of Technology, Manipal Academy of Higher Education, Manipal, 576104 Karnataka India

**Keywords:** Self-recuperation, Lightweight LSTM, Computational cost, Real-time feedback, Engineering, Health care, Mathematics and computing

## Abstract

In the modern era of working, Musculoskeletal Disorders (MSDs) are increasing drastically. One of the leading causes of MSD is Low Back Pain (LBP). Patient health monitoring technology is paramount to the investigators, enabling remote recovery services via cutting-edge technologies that lower the barrier between clinicians and patients. This work provides a low-cost, efficient, and user-friendly visual capture recovery system for the administration of Low Back Pain (LBP). This study proposes a unique computer vision and deep learning method for remotely monitoring patients’ joint angles during physiotherapy rehabilitation. A single long-short term memory layer with 64-unit lightweight model with dense neurons was used to identify the correct postures for LBP recovery exercises in real-time video. The proposed system exploits a 3D human skeleton representation for calculating angles on three landmarks to recognize the angle deviations and classify the nine LBP recuperation exercise poses with high cross-validation accuracy, low computational cost, real-time exercise correction feedback, and minimal latency to process frames. The suggested approach successfully predicts and provides feedback on LBP exercise postures from real-time video feeds captured by common RGB cameras, without additional hardware or specialist cameras, thereby improving the quality of life for people around the globe.

## Introduction

Pain in the lower back is most often caused by physical movement or by irregular standing or sitting postures. Over 90% of the population will experience this problem at some point. Most countries have between 4 and 6% economic loss in Gross Domestic Product (GDP) due to personnel suffering work-related health issues. Musculoskeletal disorders affect 1.71 billion people worldwide, according to the World Health Organisation (WHO)^1^. One recent research project, with a sample of 100 people working more than 4 h daily on a laptop or desktop, found Work-related Musculoskeletal Disorders (WMSDs). Over 12 months, WMSD prevalence affects various body parts, including the neck, shoulders, wrists, lower back, knees, upper back, and ankles^2^. Musculoskeletal discomfort is extremely prevalent, affecting millions of people worldwide. Work-related disorders are most effectively treated with exercise. It’s commonly believed that working out or moving a sore muscle can make the pain worse.

However, studies show that light exercise within your pain threshold can hasten your recovery. The study of EEG^3^, EMG^4,5^, multisensory-based^6^, and sEMG^7^ biological signals helps identify hybrid feature extraction for movement task classification. Most existing studies on human action recognition have used EEG signal analysis within a deep learning framework^8–11,58^. A bi-model AI fusion framework integrated with physiological signals, combined with an attention mechanism and LSTM, to capture the temporal dependencies to improve classification^12^.

Rehabilitation exercises are crucial in helping people regain their mobility, strength, and function after an injury or while managing a chronic disease^13–17^. Artificial Intelligence (AI) -based effective rehabilitation exercise detection has emerged as a viable technique for improving the quality and efficiency of rehabilitation programs. The AI-assisted system provides more precise feedback on the correctness of patients’ exercise poses. We deliberated the important research findings from the literature, rehabilitation using Microsoft Kinect cameras offers precise measurement of body joint angles^18^ using pose estimation techniques and excellent movement recognition. However, the implementation is costly and requires a stand-alone system. This particular product is not portable. Methods based on OpenPose and MediaPipe have been widely adopted for extracting skeletal keypoints from RGB images due to their real-time capabilities and ease of deployment. However, these models typically require large-scale datasets and substantial computational resources, which may limit their applicability in real-time, low-cost rehabilitation systems. Rehabilitation of the limbs, both upper and lower, is a key component of computer vision-based deep learning approaches^19–22^. For some rehabilitation activities, these devices typically deliver high degrees of accuracy. These systems are notoriously difficult to use in real-time. The model to be created has reduced computational complexity and processing time without compromising the accuracy^23–27^.

The important contributions of this study were,

- We propose a lightweight LSTM model for nine exercise poses. We also examine the performance of various lightweight LSTMs to select the best model for classification and to generate feedback on exercise pose correction.
- Skeletal key points in the human body were extracted through MediaPipe pose estimation, and the twelve most important body joints alone were considered to calculate the angle of a particular body joint. We extracted the ten different body joint angles from each frame as features.
- Our proposed LSTM network significantly improves accuracy and lowers computational cost against the existing literature works.

## Methodology

**Fig. 1:**
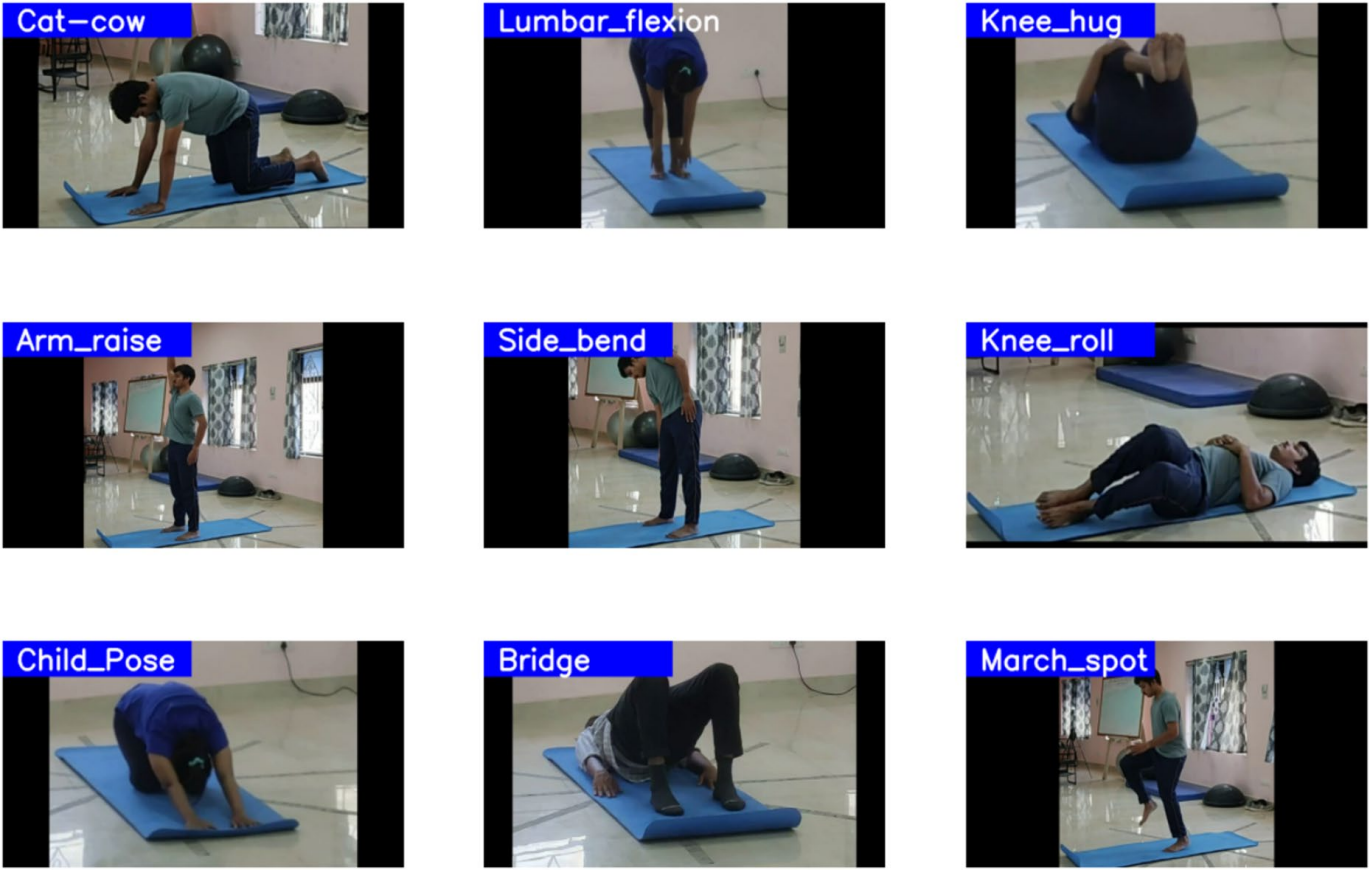
Sample LBP recuperation exercise poses.

**Fig. 2:**
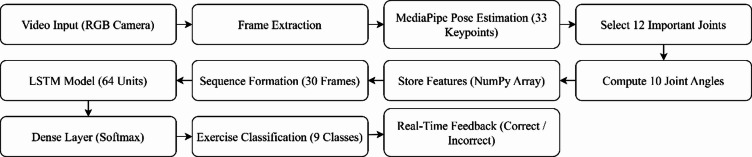
Overall system workflow.

To address the shortcomings of complex long-term data dependencies, researchers developed LSTM, an enhanced variant of RNN^28–30^, building on previous work in computer vision for human pose assessment and achieving better outcomes than are now possible. The proposed methodology analyzes video recordings of LBP-experiencing patients doing nine different physical exercises for LBP rehabilitation as mentioned in Fig. 1^36^. Once the workouts have been recorded, the body’s joints can be identified and verified. Then, an artificial intelligence-based library called MediaPipe detects the exercising individual’s body joints^31,32^. MediaPipe is a popular Google library that uses neural network principles to locate 33 human body joints^33–35^. For this study, we considered 12 important body joints to classify the aforementioned physical exercise poses. Figure 2 illustrates the overall workflow.

This section comprises the problem statement, MediaPipe Library, Participants, and the Deep learning model.

### Problem Statement

Despite the growing popularity of remote rehabilitation for the therapy of persistent LBP through exercises, there is a lack of research into their efficacy in enhancing body mobility functions among LBP patients. This article focuses on nine physical movements as shown in the Fig. 1. This figure depicts the Sample video frames collected from the participants for each LBP recuperation exercise.

### Extraction of human body key points

**Fig. 3:**
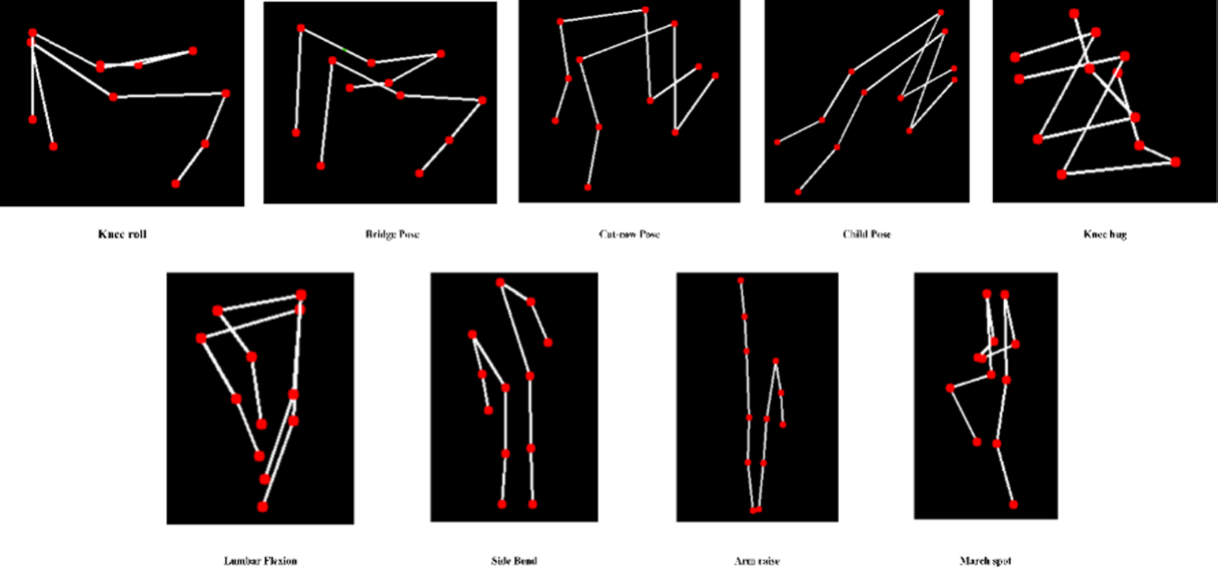
Skeletal key points for nine LBP recuperation exercise poses.

Pose estimation is an initial process for obtaining skeletal information about human poses. In this study, we also extracted 12 key skeletal points for mechanical LBP recovery exercise poses using MediaPipe pose estimation, as shown in Fig. 3.

MediaPipe was developed by Google to detect human skeletal keypoints for assessing a person’s physical pose. Ten angles are the key features calculated from 12 key points extracted from MediaPipe. We have trained our model with these angle features. Since our study aims to develop self-recuperation, we have used MediaPipe to monitor a single-person exercise pose. This selection was based on the biomechanical relevance of these joints to low back pain (LBP) rehabilitation exercises, where movements primarily involve upper limb coordination, trunk stability, and lower limb support.

To reduce computational complexity, only 10 joint-angle features are used. As a result, instead of collecting 33 landmark features from MediaPipe, only 12 important landmark features were collected to assess the 10-joint angles as input features for model training^37^. It enables the model to process faster, consuming low memory usage, and improves the real-time performance. Table 6 presents a selection of 33 landmark features and 10-joint angle features, along with validation outcomes. The results demonstrate that the proposed feature representation achieves substantially lower latency and computational cost while maintaining high classification accuracy.

### Skeleton-based LSTM network architecture

This research aims to address the scarcity of high-quality training resources by proposing a modified recurrent neural network for real-time recognition of LBP rehabilitation exercise poses. It has been shown that skeleton-based LSTM models can be used to create an intelligent LBP recuperation monitoring system that takes as input videos of sample subjects and overlays key landmarks on the human body in a visual display. The raw video of all the exercise poses performed by the user was captured through a mobile or webcam. Important twelve body joint key points were extracted using the MediaPipe pose estimation technique. Using these key points, ten key angles were calculated and stored in a numpy array format. This numpy array data was fed to the LSTM network for classifying exercise pose and providing feedback on correcting the pose in the display.

The skeletal data collected from the raw video were extracted using MediaPipe. The joint coordinates over the time of a sequence are represented as, $\:{\left\{{\boldsymbol{X}}_{t}\right\}}_{t=1}^{T}$ where, $\:{\boldsymbol{X}}_{t}\in\:{\mathbb{R}}^{N\times\:D}$. $\:t$ represents the skeleton joint positions over time, $\:N$ represents the number of joints, and $\:D$ is the dimensionality of the joint coordinates^38^. After collecting the skeleton information and joint coordinates of the human body, the angle deflection for each exercise pose was calculated. These are the input features for training our deep learning model. We preferred to calculate and collect the ten body joint angles of the human body. The body joint angle is calculated based on the three body joint coordinates. For example, calculating the elbow angle requires three body coordinates: wrist, elbow, and shoulder. It is represented as, $\:\boldsymbol{W}=\left({\boldsymbol{x}}_{\boldsymbol{w}},{\boldsymbol{y}}_{\boldsymbol{w}},{\boldsymbol{z}}_{\boldsymbol{w}}\right);\boldsymbol{E}=\left({\boldsymbol{x}}_{\boldsymbol{e}},\:{\boldsymbol{y}}_{\boldsymbol{e}},\:{\boldsymbol{z}}_{\boldsymbol{e}}\right);\boldsymbol{S}=({\boldsymbol{x}}_{\boldsymbol{s}},\:{\boldsymbol{y}}_{\boldsymbol{s}},\:{\boldsymbol{z}}_{\boldsymbol{s}})$. The two vectors are formed using the following Eq. 1 to calculate the angle.

1 $$\:{\boldsymbol{V}}_{1}=S-E=\left({x}_{s}-{x}_{e},\:{y}_{s}-{y}_{e},\:{z}_{s}-{z}_{e}\right);\:{\boldsymbol{V}}_{2}=W-E=\left({x}_{w}-{x}_{e},\:{y}_{w}-{y}_{e},\:{z}_{w}-{z}_{e}\right)$$

The vector dot product of $\:{\boldsymbol{V}}_{1},\:{\boldsymbol{V}}_{2}$ and the magnitude of $\:\Vert{\boldsymbol{V}}_{1}\Vert,\:\Vert{\boldsymbol{V}}_{2}\Vert$ determined to calculate the arctan of a particular body joint of humans. The joint angle is calculated using the following Eq. 2:

2 $$\:{\theta\:}_{elbow}=arctan\left(\frac{{\boldsymbol{V}}_{1}.{\boldsymbol{V}}_{2}}{\Vert{\boldsymbol{V}}_{1}\Vert\:\Vert{\boldsymbol{V}}_{2}\Vert}\right)$$

**Fig. 4:**
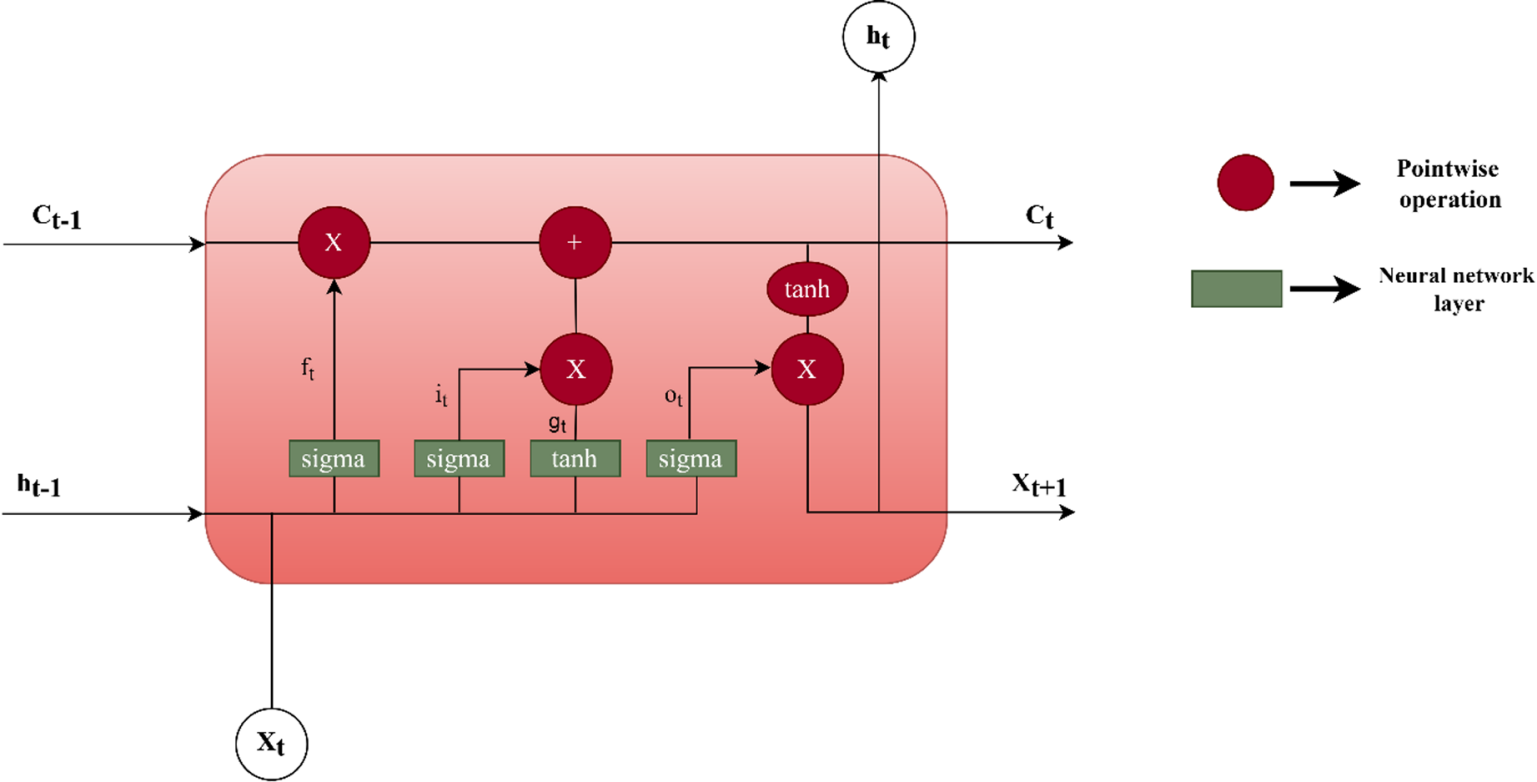
Single LSTM unit.

The pre-processed input data $\:{\boldsymbol{X}}_{t}\:$of the LSTM network contains, $\: X_{t} = [\theta \:_{{wrist,\:left}}^{t} ,\theta \:_{{elbow,\:left}}^{t},$ $\: \theta \:_{{shoulder,\:left}}^{t} ,\theta \:_{{hip,\:left}}^{t},$ $\:\theta \:_{{knee,\:left}}^{t} ,\theta \:_{{wrist,\:right}}^{t},$ $\: \theta \:_{{elbow,\:right}}^{t} ,\:\:\theta \:_{{shoulder,\:right}}^{t},$ $\:\theta \:_{{hip,\:right}}^{t} ,\theta \:_{{knee,\:right}}^{t} ]$, *‘t’* represents the angle deviations of ten joints concerning the varying time. The single unit of the LSTM network is depicted in Fig. 4.

An LSTM relies on a memory cell that can recall or forget data in discrete chunks over extended periods^39,40^. Each part of this memory cell interacts with the others in a carefully orchestrated manner. These are the major parts of an LSTM cell:

The cell state (Ct) in an LSTM is updated using the following Eq. 3:

3 $$\:{C}_{t}={f}_{t}*{C}_{t-1}+{i}_{t}*{g}_{t}$$

The computation of the input gate $\:{i}_{t}$ can be expressed in Eq. 4:

4 $$\:{i}_{t}=\sigma\:({W}_{i}*\left[{X}_{t},\:{h}_{t-1}\right]+{b}_{i})$$

The forget gate selects cell state data to delete. It derives a value between 0 and 1 for each cell state element. The computation of the forget gate $\:{f}_{t}$ can be expressed in Eq. 5:

5 $$\:{f}_{t}=\sigma\:({W}_{f}*\left[{X}_{t},\:{h}_{t-1}\right]+{b}_{f})$$

The output gate can be expressed as in Eq. 6:

6 $$\:{o}_{t}=\sigma\:({W}_{o}*\left[{X}_{t},\:{h}_{t-1}\right]+{b}_{o})$$

The mathematical expression for the hidden state is given in Eq. 7:

7 $$\:{h}_{t}={o}_{t}*\mathrm{t}\mathrm{a}\mathrm{n}\mathrm{h}\left({C}_{t}\right)$$

### Calculation of parameters in each LSTM layer

A neural network’s efficiency and performance are closely tied to its memory usage. A model’s inference time, energy usage, and scalability might all suffer if it requires a lot of memory. When you have a firm grasp of the memory footprint, you can tweak the model’s architecture to use less RAM and run more efficiently^41^. We need to know how much memory it uses to select the right model, deploy it correctly, and optimize it to maximize your resources while keeping your system stable. The mathematical expression for our proposed model is expressed in Eq. 8.

8 $$\:Number\:of\:LSTM\:Parameters=4\times\:\left(\right(x+h)\times\:h+h)$$

where x - input dimension; h - number of LSTM units. The body joint angles, such as Wrist, Elbow, Shoulder, Hip, and Knee, on both the right and left sides, are the ten key landmarks used as features for this work. LSTM Layer: 4× ((10 + 64) ×64 + 64) = 19,200, input dimension = 10, hidden layer = 64; Dense output Layer: 64 × 9 + 9 = 585, input dimension = 30,10, output layer = 9. The overall memory footprint of our ensemble LSTM with 10 body joint angles as features is 77.79 KB, with 1206 FLOPs.

A non-linear activation function produces a smooth, symmetrical output by squashing input values to the range − 1 to 1. The tanh activation function formula is described in Eq. 9^42^.

9 $$\:tanhx=\:\frac{{e}^{x}-{e}^{-x}}{{e}^{x}+{e}^{-x}}\:\:$$

A SoftMax activation function is described as a mathematical equation in Eq. 10.

10 $$\:{\sigma\:\left(z\right)}_{i}=\frac{{e}^{{z}_{i}}}{\sum\:_{j=1}^{K}{e}^{{z}_{j}}}$$

The subsequent 30 frames are used to poll this layer for its output and make the prediction. As shown in Eq. 11, the model quickly converges to incorporating a momentum component and a scaling term^43^.

11 $$\:{\theta\:}_{new}=\:{\theta\:}_{old}-\eta\:.\widehat{m}.\phi\:\sqrt{\widehat{s}+\epsilon}$$

where $\:{\theta\:}_{new}$ and $\:{\theta\:}_{old}$ represent the updated and baseline weight values, $\:\eta\:$ represents the learning rate, $\:\widehat{m}\:$represents the momentum term, $\:\widehat{s}$ represents the scaling term, $\:\epsilon$ represents the smoothing term to prevent a zero-division error, and $\:\phi\:$ represents the element-wise division operation. Categorical cross-entropy, a prominent loss function for multiclass classification tasks, is employed here. The loss function for a categorical cross-entropy^44^ is shown mathematically in Eq. 12.

12 $$\:{E}_{cc}=-\frac{1}{N}\sum\:_{i=1}^{N}\sum\:_{c=1}^{c}\left({p}_{ic}\mathrm{l}\mathrm{o}\mathrm{g}\left({y}_{ic}\right)\right)$$

where, $\:{E}_{cc}$ represents categorical cross entropy, c category and i training pattern, N represents the number of pairs available in training data, $\:{p}_{ic}$ represents the binary indicator for detecting the relation between the i and c terms. $\:{y}_{ic}$ represents the predicted probability distribution.

## Procedure

Frames from video sequences are fed into the proposed system in real-time. The result would be the optimal exercise position for recovery from low back pain. Keypoint extraction and pose prediction are the two major components of the system. The key points extraction phase aims to pinpoint the location of relevant key points relative to the user. Model architecture and posture classification are both established during the pose prediction stage. Stance prediction is the last step in which the user visualizes the stance.

### Data collection

The dataset was acquired using multiple imaging devices, including a handheld RGB camera, webcams, and a smartphone camera. Recordings were performed from four different viewpoints (35°, 135°, 225°, and 315°) to capture diverse perspectives. To ensure consistency, participants were positioned at a fixed distance of approximately 1.5–2 m under controlled indoor lighting conditions. A total of 108 video samples were collected, covering nine rehabilitation exercises performed by sixteen participants. The participants’ demographic details are represented in Table 1.

**Table 1:** Demographic and clinical distribution of study participants.

	Categories	Participants data
**Gender**	Male	12
	Female	4
**Age**	Mean	25.5
	Standard Deviation	3.77

**Fig. 5:**
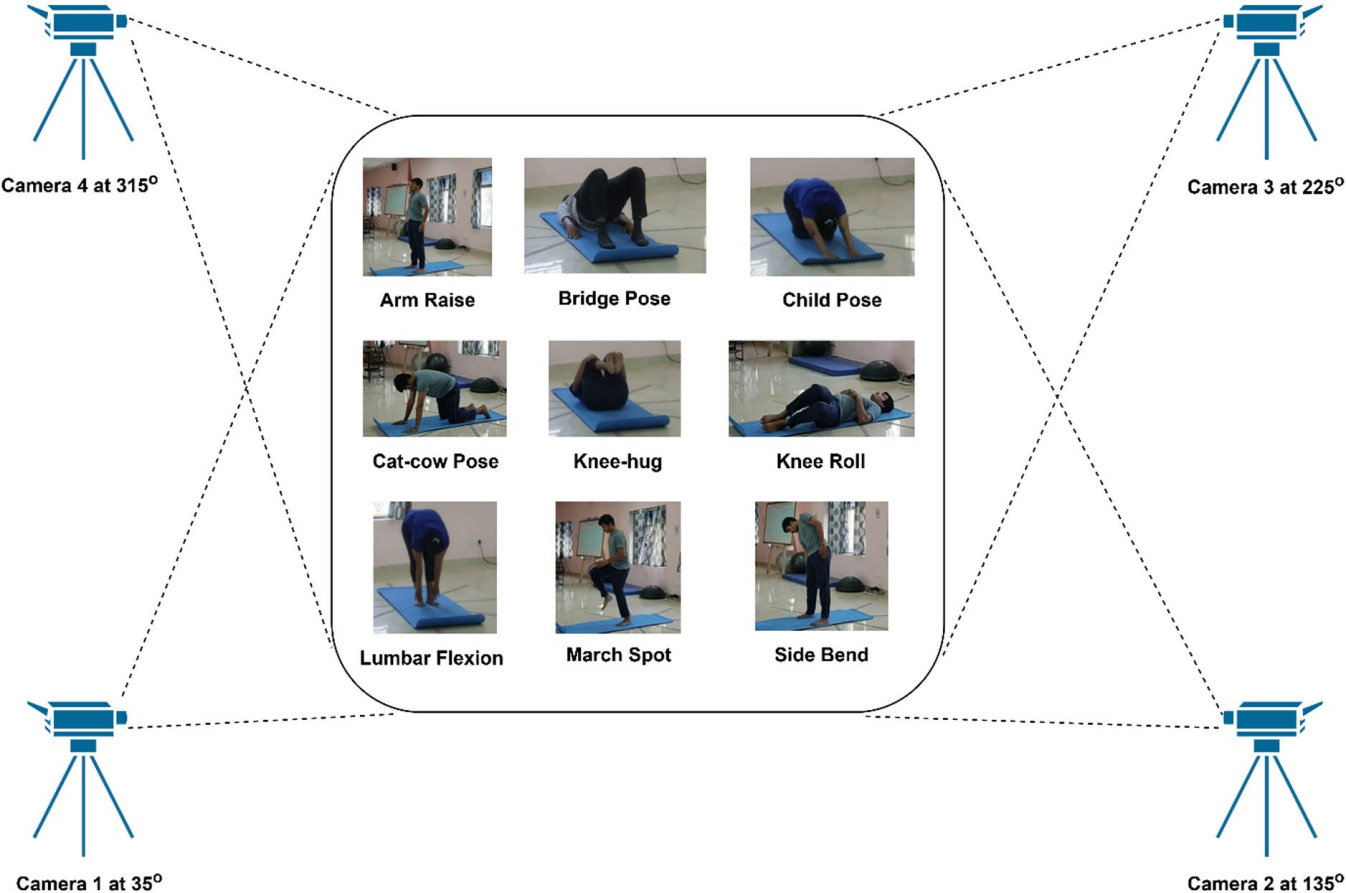
Camera Setup for Data Collection.

The data collection was conducted in an indoor environment with uniform lighting conditions to minimize shadows and illumination variability. A relatively plain background was used to reduce visual noise and improve the robustness of MediaPipe-based keypoint extraction. These controlled conditions ensured reliable extraction of skeletal landmarks and improved the consistency of the dataset. Participants were selected from four distinct perspectives, and the cameras were positioned at appropriate angles. Figure 5. Shows the camera setup for data collection.

Combining data from multiple camera perspectives can improve the precision of the analysis. These datasets can strengthen the generalizability and robustness of AI models by providing diverse perspectives. Table 2 shows the dataset size collected in the controlled environment.

**Table 2:** Summary of dataset.

S. No.	Parameters	Dataset Detail
**1**	No. of frames	43,200 frames
**2**	Size of dataset	4.74GB

### Experimental setup

**Fig. 6:**
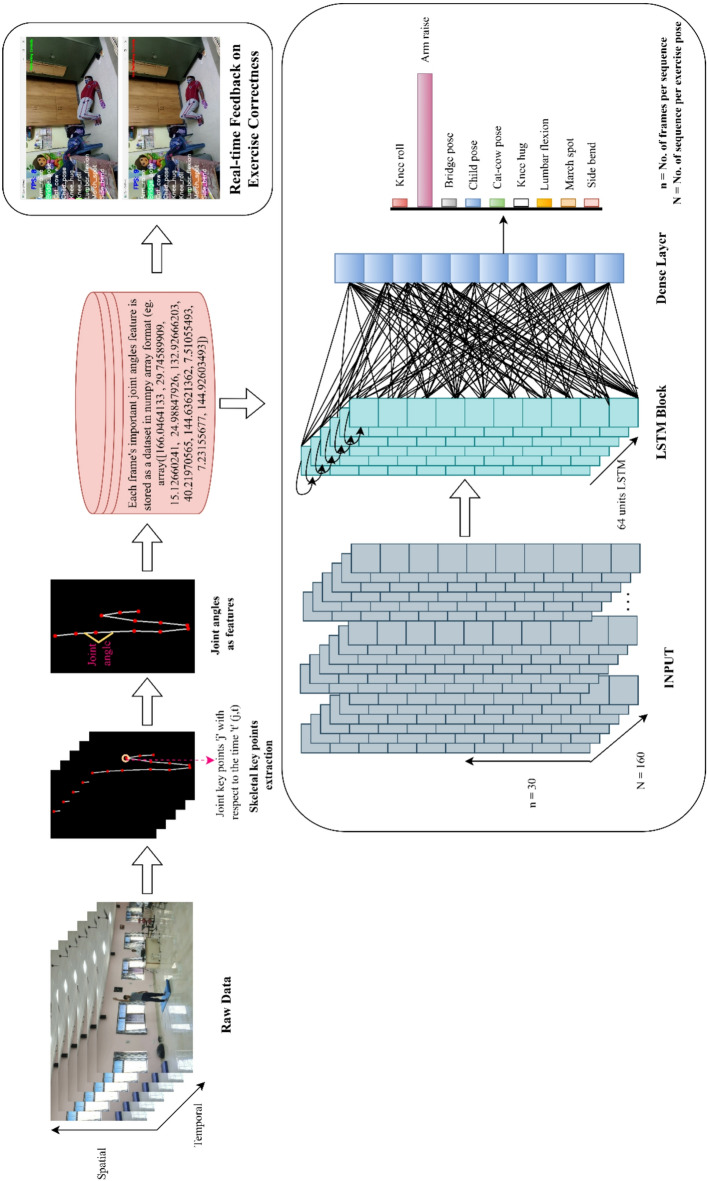
Structure of system process flow.

In this study, data were collected from sixteen volunteer participants who could perform rehabilitation exercises correctly under controlled conditions. Who can perform the exercises properly, in line with the Sussex National Health Service (NHS)^36^. Twelve male and four female participants have shown interest. The mean age of the participants is 25.5 ± 3.77. Detailed information about the experiment was provided to each participant before they participated in the study. In the first stage, all the video’s frames are used to extract keyframes, which are then stored in a NumPy array. After transcoding the videos into a NumPy array. Each test case, with a sequence length of 160 and 30 frames, contains the coordinates of 33 key points, including 63 key points on the left and right hands. The data is split 80:20, allowing us to express the input shape for a single test case as (30, 258). Additionally, we investigated the use of deeper multi-layer LSTM architectures. However, it was observed that multi-layer configurations increased computational complexity and inference latency without a significant improvement in classification accuracy for the given dataset^37^. A single-layer LSTM architecture with 64 units was selected as an optimal trade-off between performance and efficiency. Figure 6. Shows the proposed system process flow.

We used the MediaPipe library for keypoint extraction, a Google cross-platform package that offers powerful prebuilt ML solutions for computer vision problems. Examples of key points include shoulders, elbows, wrists, knees, and other body parts that are important in creating LBP rehabilitation exercise poses. This phase uses machine learning techniques to build data structures tailored to the specific application. The contribution is made by both the efficient system creation and evaluation operations. The second step involves developing a deep learning model to accurately assign each live video to one of the nine exercises in the dataset’s accompanying video sequences. Here, an ensemble LSTM model helps comprehend the frames observed during an LBP recovery workout stance. Specifically, an LSTM is a subset of RNNs designed to learn and remember extremely long-term dependencies across extended sequences of input data. We have used a single LSTM layer, which is especially helpful for video frames or time-series data from a NumPy array.

Furthermore, the probabilities of each LBP recovery exercise position are calculated with a Softmax layer, which employs a normalized exponentiation function. The output is the pose with the highest probability. Tanh, the activation function utilized in this layer, controls how information flows by preventing cells’ gradients from disappearing or inflating as they take in and relay crucial information. We need 30 frames of data, concatenated in sequence, to generate a prediction. Empirical observations during preliminary experiments indicated that a sequence length of 30 frames provides a stable representation of exercise motion while ensuring low-latency inference. Therefore, this configuration was adopted as an optimal balance between temporal dependency modelling and computational efficiency for real-time rehabilitation monitoring. Ten key body joint angles are selected as features to classify the exercise pose based on the input visual. Hyperparameter tuning was performed using a grid search optimization strategy to systematically identify the optimal configuration of the model. LSTM neural networks have also been shown to be efficient and beneficial for certain applications. The parameter settings for prediction and feedback generation are shown in Table 3.

**Table 3:** Hyperparameter selection for the proposed model.

Parameter name	Value for the various exercise pose Dataset
Input shape of the network	30, 10
Network LSTM units	64
Learning rate	0.001
Training batches	32
Number of epochs	200

The LSTM with 64 units has an input dimension of 30 sequences of 10 body joint angle features, and a single dense layer for exercise pose classification. The entire process was developed using Python. The framework was created using Keras, Numpy, Scikit-Learn, and Tensorflow libraries. The Mediapipe program’s Windows version was chosen to run. The following are the technical characteristics of the computer used to run the Mediapipe software library to extract the train and test data modules: a built-in NVIDIA GeForce MX330 graphics processing unit and an 11th-generation Intel(R) Core (TM) i5-1135G7 CPU with 2 GB of RAM. Using polling, the prediction of the LBP rehabilitation exercise poses is performed over a sequence of 30 frames. The mode of all exercise pose predictions is considered the final prediction. This section is subdivided into three subsections: Overall system prediction accuracy vs. loss for training and testing datasets, Performance evaluation metrics, and Accuracy comparison with existing approaches. To further validate the robustness of the proposed model, 5-fold cross-validation was performed. Table 4. Shows the 5-fold cross-validation of the proposed model.

**Table 4:** 5-fold cross-validation of the proposed model.

Fold	Accuracy (%)
Fold 1	96.88
Fold 2	98.96
Fold 3	97.22
Fold 4	98.96
Fold 5	95.49
**Mean ± Std**	**97.50 ± 1.32**

## Results and discussion

By effectively enhancing human mobility to reduce the prevalence of MSD, this study provides an effective self-rehabilitation solution. Two-stream GCNs^45–47^ combined spatial and temporal features from separate GCNs, enhancing overall performance. The model achieved a cross-validation accuracy of 100%, but it is computationally expensive and requires a specialized computing platform to perform the analysis. Long-Term Recurrent Convolutional Networks (LRCN) integrate CNNs for spatial feature extraction and LSTMs for temporal sequence modelling^48^, providing a robust baseline. The spatiotemporal ConvST-LSTM-Net^49^ improved the accuracy of human action recognition. We experimented with this model on our generated dataset; it achieved 55% cross-validation accuracy. We trained this model for 200 epochs. Improving accuracy requires increasing the number of training epochs. The computational cost of this model is also high. Recovery exercise validation and recognition using a modular neural network trained via computer vision-based (OpenPose) tracking of the patient’s body joints^21^. A baseline CNN using the dense net model^54^ for predicting the correctness of exercise pose requires spatial features for each frame. This model is also computationally expensive. Because it superimposes the 18-body joint critical points, the OpenPose pose estimation method might not provide reliable workout pose predictions.

A method to categorize the action and infer its posture was suggested by Rangari et al.^50^. Currently, it is not easy to classify incorrect plank positions using the LSTM approach. Posture action classification using the OpenPose Model is performed by Hongyan Zheng, Haijun Zhang, and Hao Zhang^51^. Still, achieving strong resilience while improving accuracy and real-time pose prediction is difficult. Hang Cai^52^, an analysis of fitness motions in real-time. Only one limb’s motion can be tracked using this method. The system’s capability to track many limbs concurrently has also been enhanced. Ratnesh Prasad Srivastava et al.^53^ proposed a custom LSTM model for predicting small custom datasets but achieved an average accuracy of 92.34%. The model with a multilayer perceptron (MLP) proposed by Vivek Anand Thoutam et al.^55^ achieves low computational cost compared to other deep learning models but fails to achieve high prediction and feedback generation accuracy. A deep learning model that can recognize yoga poses in real-time from videos captured using a combination of CNN and LSTM has been developed by Swain D et al.^56^. This model is also computationally large to process the data. Only three positions were identified for the rehabilitation exercise. The experimental evaluation of a 64-unit LSTM shows that it provides the best balance between accuracy and computational efficiency. The following subsections deliberate the effectiveness measurement of performance evaluation.

### Overall system prediction accuracy vs. loss for training and testing datasets

**Fig. 7:**
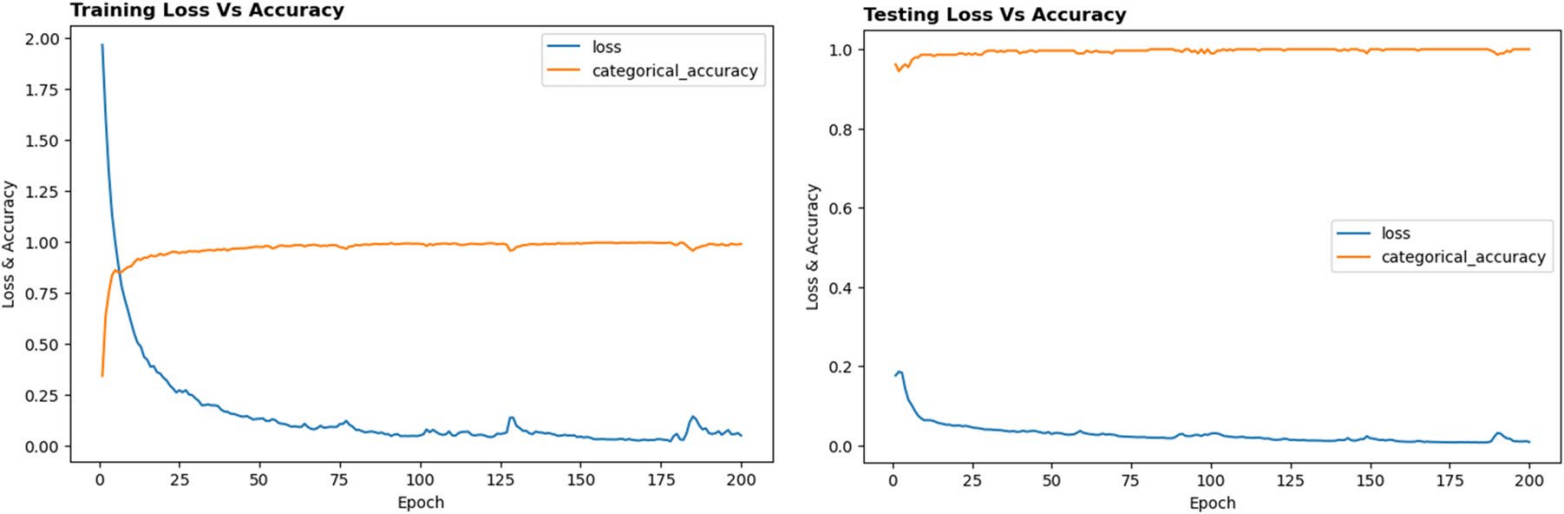
Training and Testing Loss and accuracy over each epoch.

The training and test accuracies and losses are plotted in Fig. 7. After 200 epochs, our best-trained model with 10 body joint angles as input achieved 99.2% training and 100% testing accuracy.

**Fig. 8:**
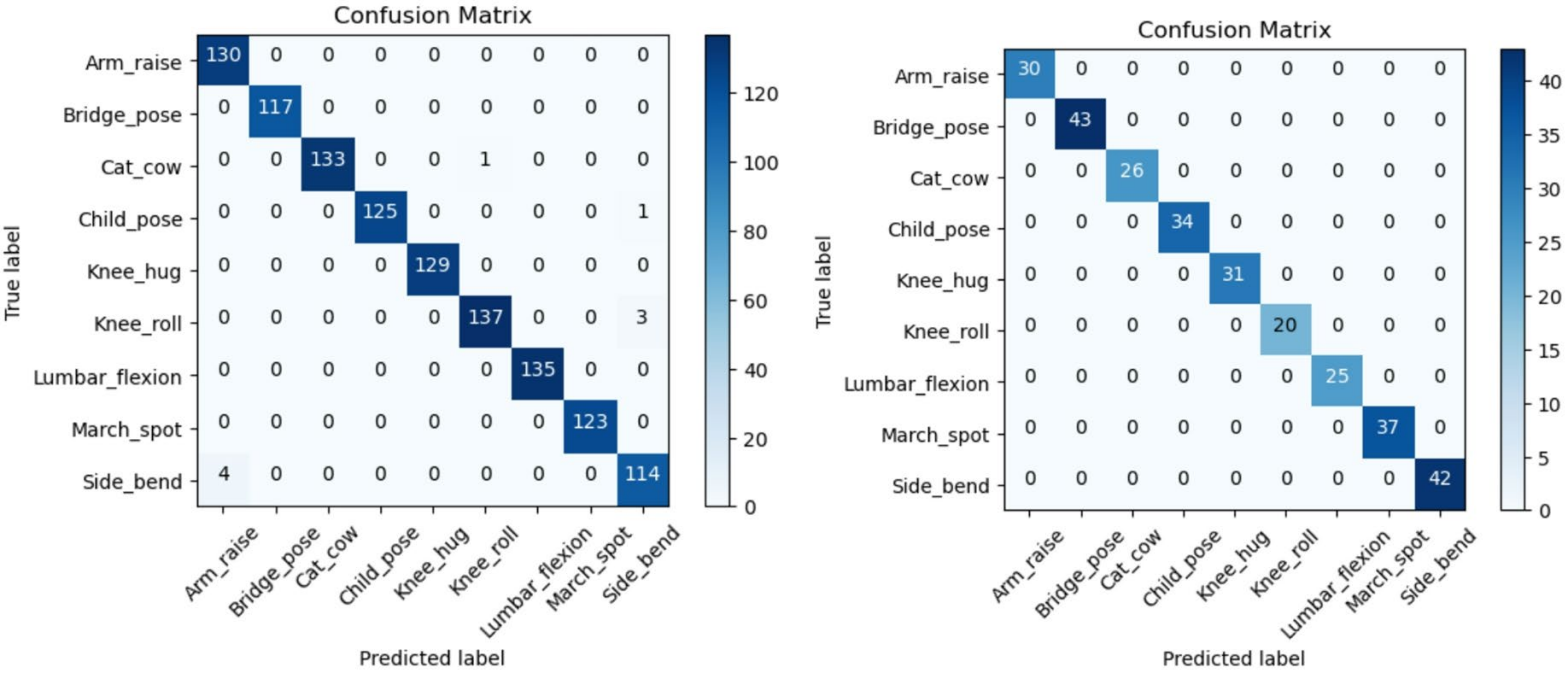
Confusion matrix for training and testing data.

The confusion matrix plays a crucial role in calculating metrics such as recall, accuracy, precision, and F1-score. It can also synthesize prediction findings for classification issues. The confusion matrices for both the training and test data of our proposed system are shown in Fig. 8.

The performance metrics of the model are calculated using the following Eqs. 13–16:

13 $$\:Accuray=\:\frac{TP+TN}{TP+TN+FP+FN}$$

14 $$\:recall=\:\frac{TP}{TP+FN}$$

15 $$\:Precision=\:\frac{TP}{TP+FP}$$

16 $$\:F1-Score=\:2\times\:\frac{Precision\:\times\:recall}{Precision+recall}$$

The classification report for the proposed system model is shown in Table 5.

**Table 5:** Classification report and its performance metrics.

Exercise Pose	Training Precision	Training Recall	Training F1-score	Testing Precision	Testing Recall	Testing F1-score
Arm_raise	0.970	1.000	0.985	1.000	1.000	1.000
Bridge_pose	1.000	1.000	1.000	1.000	1.000	1.000
Cat_cow	1.000	0.993	0.996	1.000	1.000	1.000
Child_pose	1.000	0.992	0.996	1.000	1.000	1.000
Knee_hug	1.000	1.000	1.000	1.000	1.000	1.000
Knee_roll	0.993	0.979	0.986	1.000	1.000	1.000
Lumbar_flexion	1.000	1.000	1.000	1.000	1.000	1.000
March_spot	1.000	1.000	1.000	1.000	1.000	1.000
Side_bend	0.966	0.966	0.966	1.000	1.000	1.000
Accuracy			0.992			1.000

### Accuracy comparison with existing approaches

**Fig. 9:**
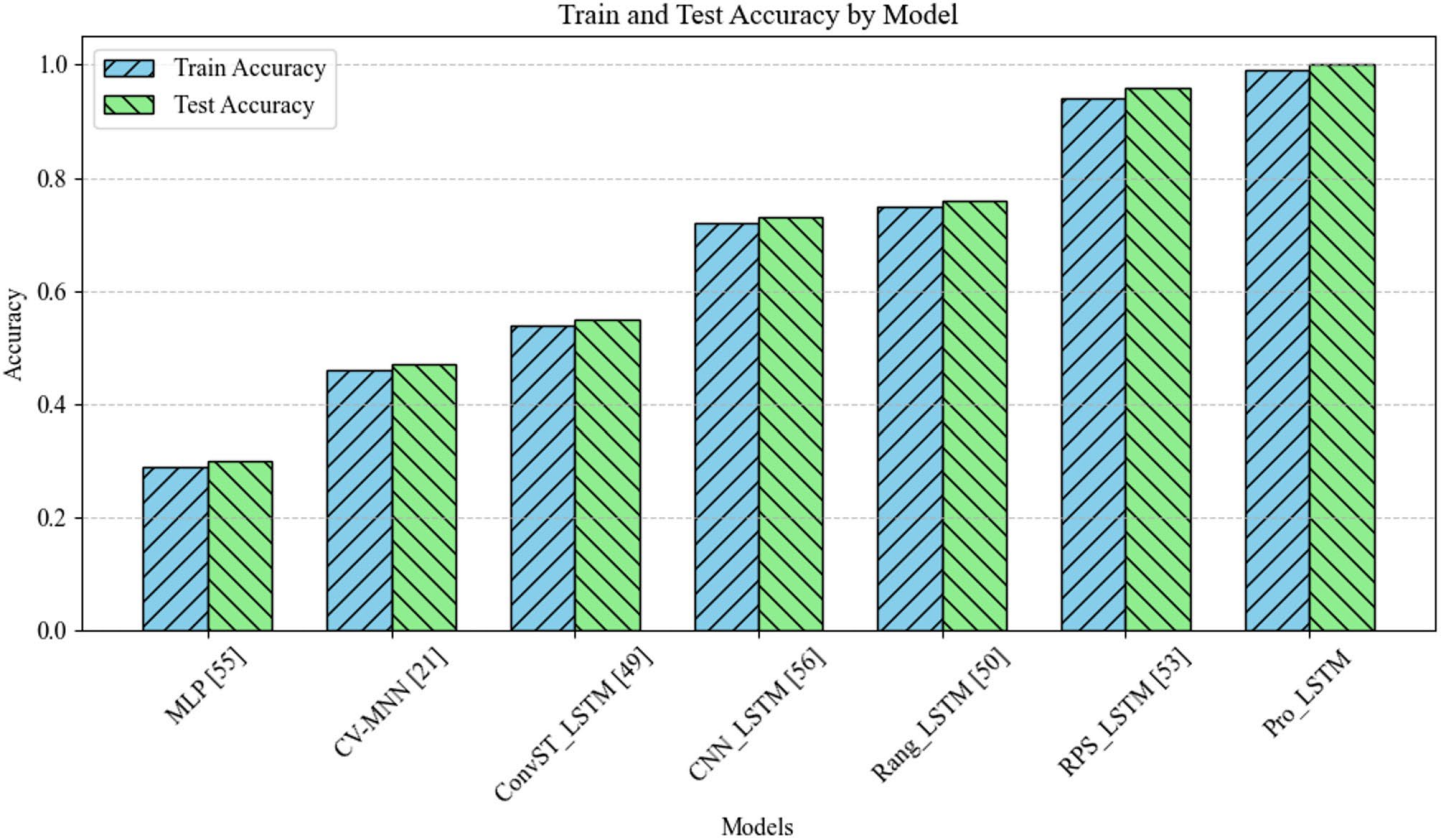
Comparison of our proposed model with existing models.

We performed a thorough evaluation of the accuracy of several models to assess their performance for skeleton-based workout pose recognition. Our proposed lightweight LSTM is one of the models compared with the Computer Vision Modular Neural Network^21^, ConvST-LSTM^49^, Modified LSTM^50^, custom LSTM^53^, Multilayer Perceptron (MLP)^55^, and 1-D CNN with LSTM^56^. Figure 9 compares the training and test accuracies of various existing model with proposed model.

The proposed model achieved the highest accuracy of 99.2% with training data and 100% for testing data, outperforming the MLP, Modular neural network, and various customized LSTM models.

### Sample outcome of the proposed system

**Fig. 10:**
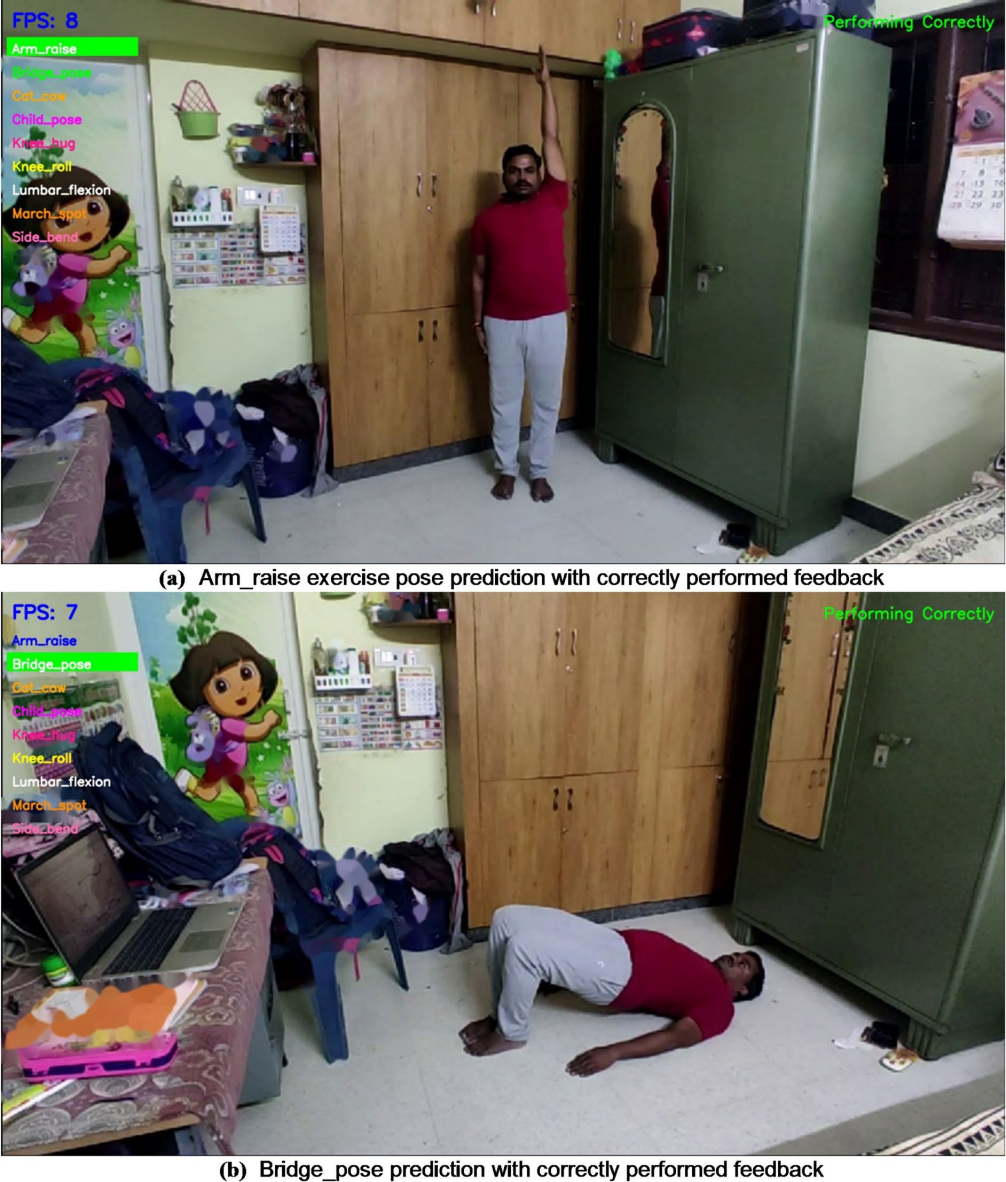

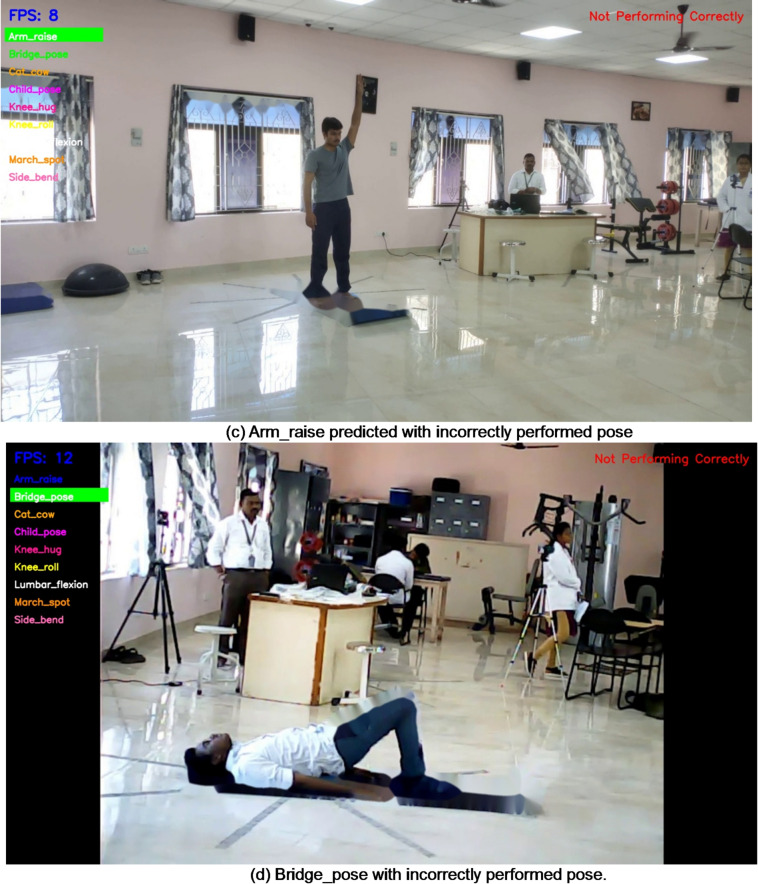
Sample outcomes of exercises Arm_raise and Bridge_pose with correctly performed and incorrectly performed poses.

The user or trainer is often given a visual representation of the workout posture prediction results. These visual representations might be as simple as superimposing the estimated postures on top of the video feed, as complex as emphasizing correct or erroneous movements, indicating joint angles, or developing graphical representations of the workouts. Sample outcomes from the proposed system are shown in Fig. 10 below.

### Ablation experiments

**Fig. 11:**
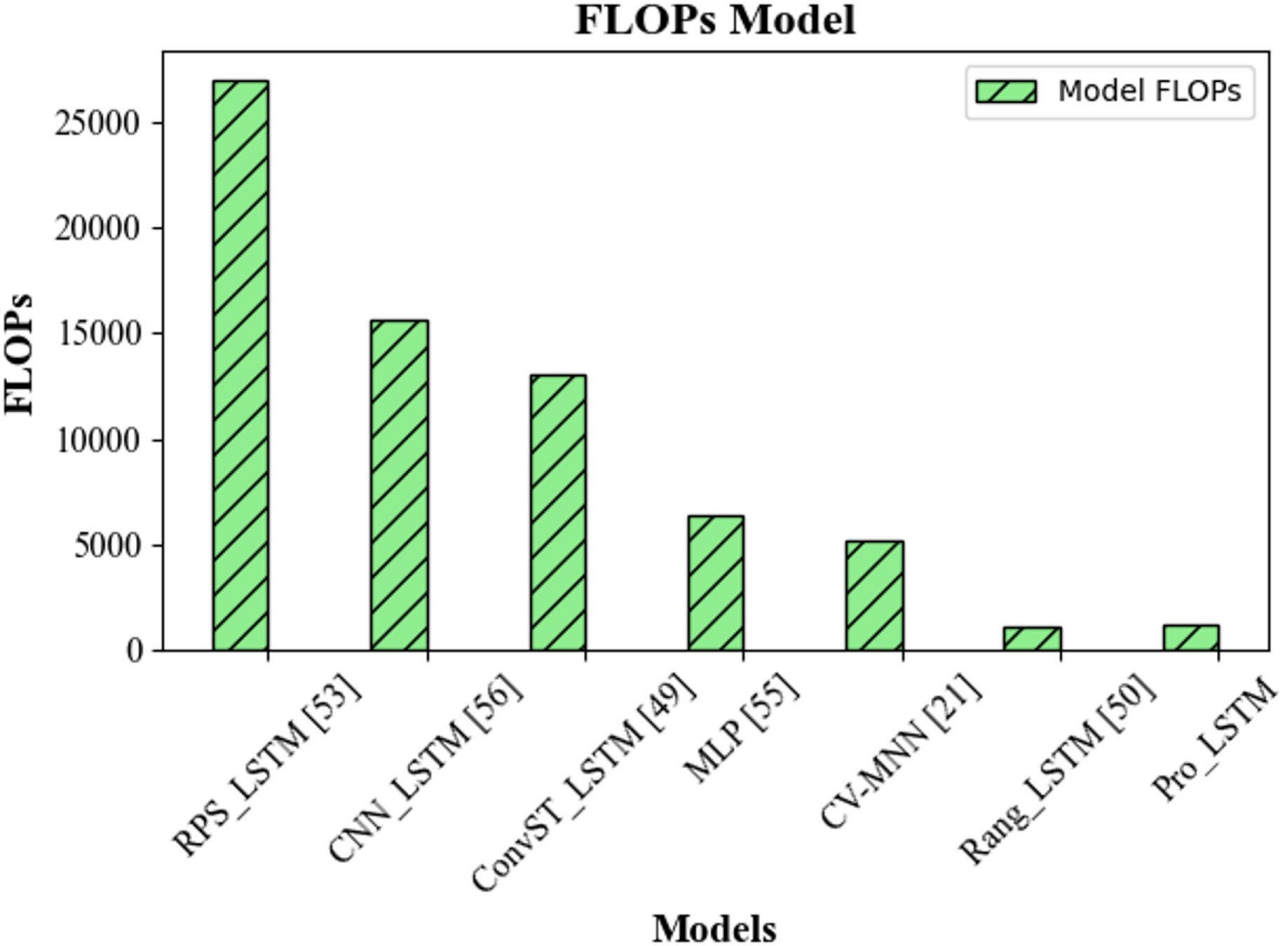
A detailed comparison is conducted to assess the computational complexity of the proposed model relative to state-of-the-art methods.

In many real-time and latency-sensitive applications, detecting and reducing latency in deep learning models is critical. To confirm that the suggested skeleton-based LSTM accurately predicts low computational cost for the various exercise poses in LBP management using body joint angles as features, we conducted an ablation experiment across various existing proposed models using our dataset. The computational complexity of various models is plotted in Fig. 11.

Performance in scientific computations, simulations, and machine learning tasks is often measured in FLOPs (floating-point operations per second). Counting the floating-point operations performed by each layer and operation in a neural network or by a specific operation yields the total number of FLOPs needed by the network or by the operation. The mathematical expression for the neural network FLOPs^57^ is represented in Eq. 17.

17 $$\:FLOPs=2\times\:{H}_{o}\times\:{W}_{o}\times\:{C}_{o}\times\:({K}_{h}\times\:{K}_{w}\times\:{C}_{i}+1)$$

The LSTM layers with skeleton features can be expressed in Eq. 18.

18 $$\:FLOPs\:per\:LSTM\:cell=4\times\:(2\times\:{D}_{h}\times\:{D}_{i}+2\times\:{D}_{h}^{2}+3\times\:{D}_{h})\:$$

Where, $\:{D}_{i}$ – Dimension of input features, $\:{D}_{h}\:$- Dimension of the hidden state.

**Fig. 12:**
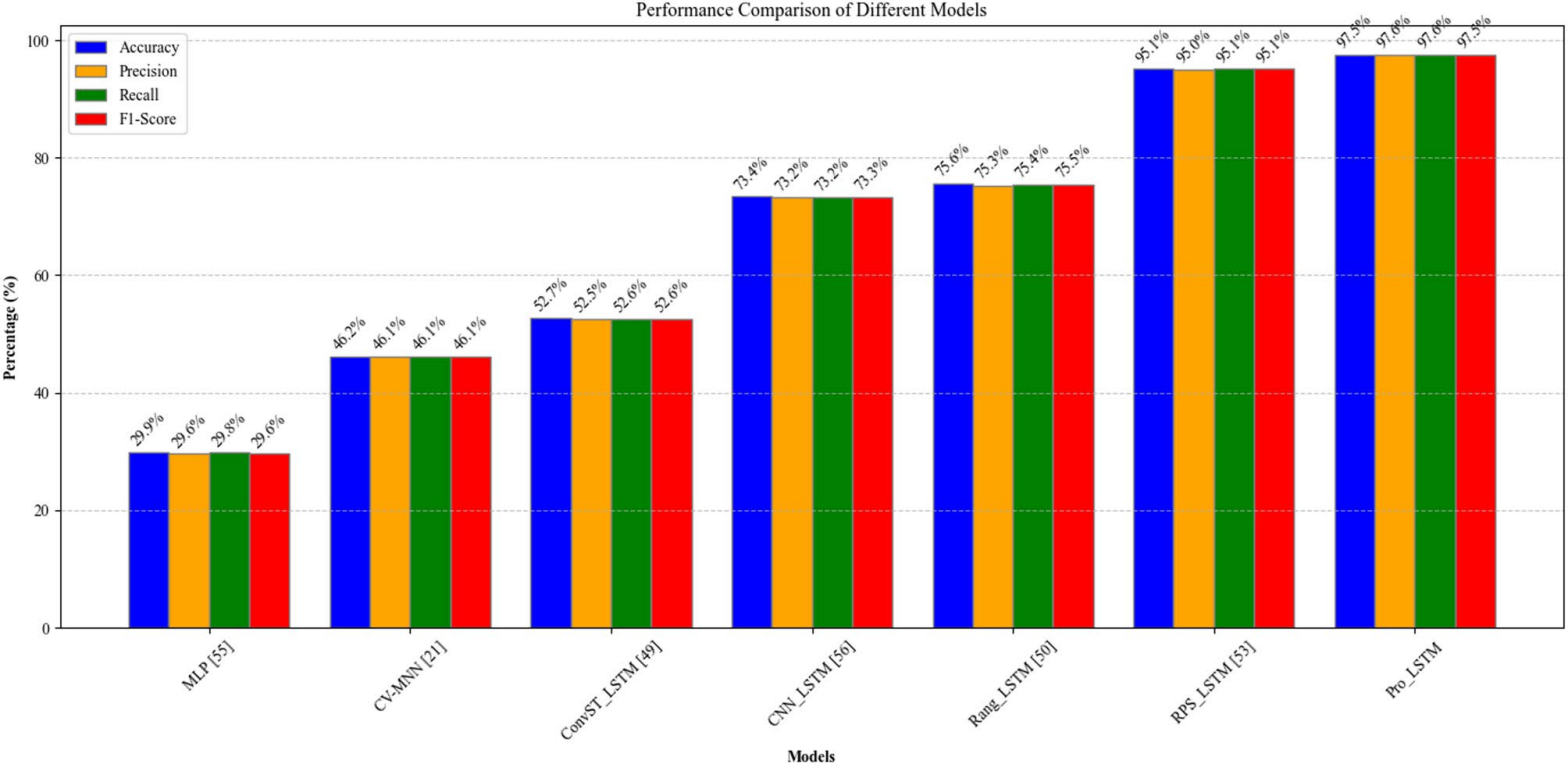
Comparison of existing models with the proposed model’s performance metrics.

We have also evaluated the performance comparison metrics accuracy, precision, recall, and F1-score for existing and proposed models. Figure 12 shows the comparison analysis of performance metrics.

The 5-fold cross-validation was performed to ensure the generalization and robustness of the model. An average accuracy of the proposed model is 97.50%, indicating consistent performance. Other important metrics, such as precision, recall, and F1-score, were assessed and achieved 97.62%, 97.60%, and 97.53%, respectively. In many real-time and latency-sensitive applications, detecting and reducing latency in deep learning models is critical. Wall time is the total elapsed time, including delays at the system level, I/O activities, and resource wait times. This metric reflects the time taken from the beginning to the end of a process. The time the central processing unit (CPU) spends executing a program’s instructions is quantified. It doesn’t factor in things like sitting about or performing system tasks that have nothing to do with running the actual program. The comparison of latency, CPU time, and Wall time of our proposed approach with two different features is described in Table 6.

**Table 6:** Comparison of time complexity between 33 landmark features vs. 10 key angle features.

Parameters measured	33 landmarks as input features	Ten important body joint angles as features
Latency in (sec)	777.9	0.08
Wall Time in (sec)	338	155
CPU Time in (sec)	777	265

Based on the observation from the Table 6, we can conclude that the task or model associated with the LSTM with 33 landmarks features from the MediaPipe library of results had significantly higher latency, consumed more CPU time, and took a longer wall time to complete compared to the LSTM with ten essential body joint angles as features with MediaPipe of results. This suggests that the LSTM with 258 key points was more complex, resource-intensive, or time-consuming than the LSTM with ten key body joint angles.

**Fig. 13:**
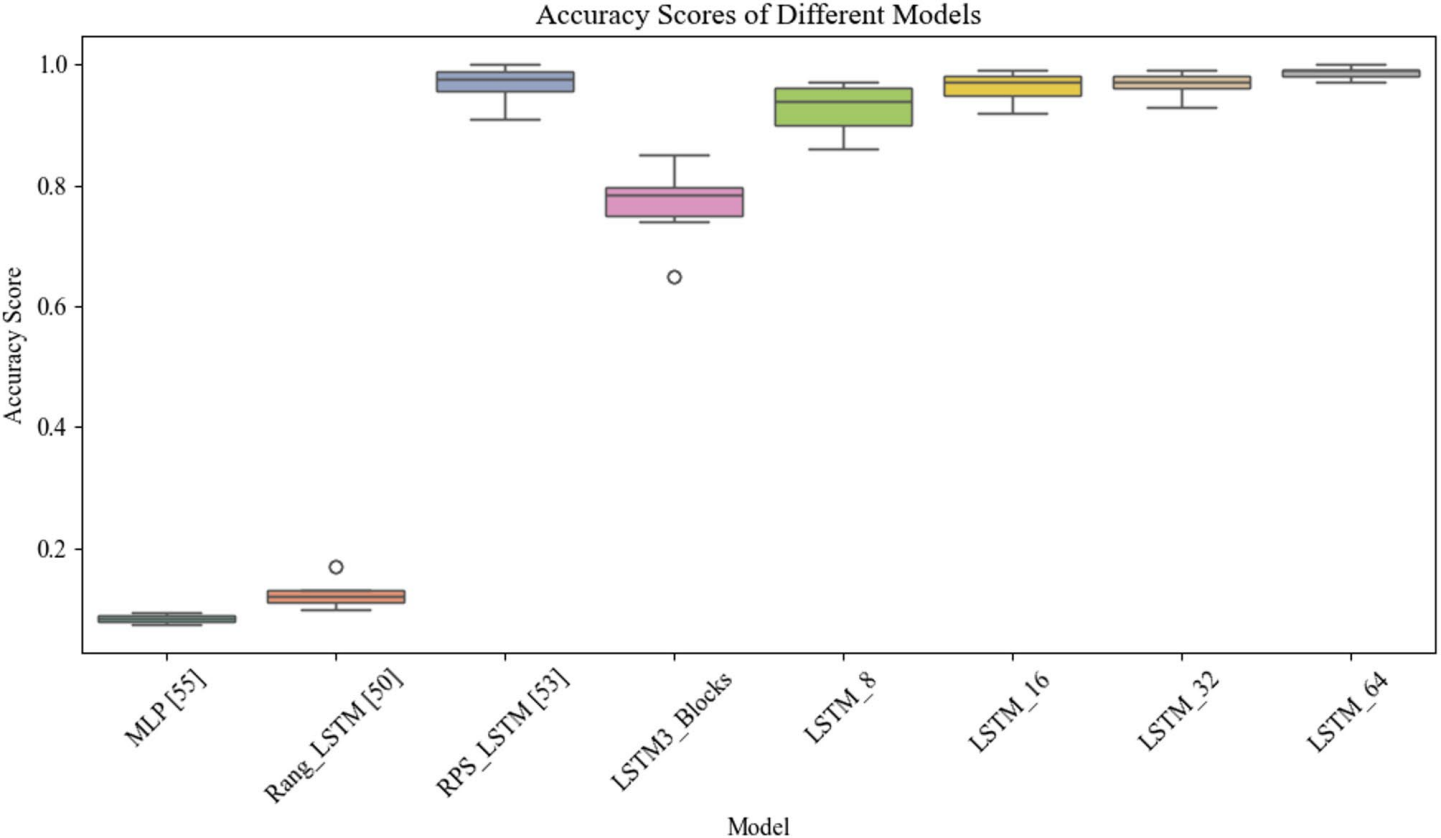
Comparison of the mean accuracy score for the proposed model.

Figure 13 compares the accuracy scores obtained by different machine learning models, in order: MLP, Rang_LSTM, RPS_LSTM, LSTM3_Blocks, LSTM_8, LSTM_16, LSTM_32, and LSTM_64. The distribution of accuracy scores across several trials is shown via a box plot, which also depicts each model’s performance. Each model’s performance consistency and variability can be clearly shown in the box plot, which includes the outliers, median accuracy, and interquartile range (IQR). By comparing the findings, it is easy to see which models provide the most consistent and accurate outcomes.

## Conclusion

Patients undergoing physiotherapy rehabilitation exercises can have their joint angles remotely monitored using a novel computer vision and deep learning approach, as shown in this study. Recognizing the proper positions for low back pain rehabilitation workouts in real-time video was accomplished using a single 64-unit LSTM model equipped with dense neurons. It uses ten key joint angle features to classify exercises with minimal latency per frame, high cross-validation accuracy, low computational cost, and real-time exercise correction feedback. In comparison to MLP, Modular neural network, and several customized LSTM models, the suggested model outperformed them all, achieving a maximum accuracy of 99.2% on training data and 100% on testing data. The LSTM_64 model’s median accuracy is particularly high compared to existing models. The evaluation shows that our model outperforms the existing models across all the performance metrics. Despite achieving high classification accuracy, this study has certain limitations. Consequently, the generalizability of the proposed system to clinical populations remains to be validated. Another limitation of this study is that the system was evaluated under controlled environmental conditions. Real-world condition scenarios, such as different lighting environments, background clutter, and partial occlusion of body joints, have not been fully investigated. These factors may influence the accuracy of pose estimation and classification performance. Future work will focus on collecting data from LBP patients and conducting clinical evaluations to assess the system’s effectiveness. Exploring advanced LSTM variants such as ResLNet (Residual LSTM Network)^12^, Attention-Bidirectional Peephole Long Short-Term Memory (Att-BiPLSTM)^59^, Peephole LSTM^60^, Nested LSTM^61^, and other optimized recurrent architectures to further enhance model performance while maintaining real-time constraints and exploring adaptive and variable-length sequence modelling to further optimise system performance.

## Data Availability

The datasets generated and/or analysed during the current study are not publicly available due to the data being part of an ongoing study. Requests to access the datasets should be directed to the corresponding author.
